# Consumer Preference and Attitude Regarding Online Food Products in Hanoi, Vietnam

**DOI:** 10.3390/ijerph15050981

**Published:** 2018-05-14

**Authors:** Anh Kim Dang, Bach Xuan Tran, Cuong Tat Nguyen, Huong Thi Le, Hoa Thi Do, Hinh Duc Nguyen, Long Hoang Nguyen, Tu Huu Nguyen, Hue Thi Mai, Tho Dinh Tran, Chau Ngo, Thuc Thi Minh Vu, Carl A. Latkin, Melvyn W.B. Zhang, Roger C.M. Ho

**Affiliations:** 1 Institute for Preventive Medicine and Public Health, Hanoi Medical University, Hanoi 100000, Vietnam; kimanh.yhdp@gmail.com (A.K.D.); bach.jhu@gmail.com (B.X.T.); lethihuong@hmu.edu.vn (H.T.L.); duchinh@hmu.edu.vn (H.D.N.); huemt93@gmail.com (H.T.M.); 2 Bloomberg School of Public Health, Johns Hopkins University, Baltimore, MD 21205, USA; chau.ngo52@gmail.com (C.N.); carl.latkin@jhu.edu (C.A.L.); 3 Vietnam Young Physicians’ Association, Hanoi 100000, Vietnam; huutu85@gmail.com; 4 Institute for Global Health Innovations, Duy Tan University, Da Nang 550000, Vietnam; 5 Department of Nutrition and Food Safety, Hanoi Medical University, Hanoi 100000, Vietnam; dothihoa1954@yahoo.com; 6 Department of Public Health Sciences, Karolinska Institutet, SE-171 77 Stockholm, Sweden; longnh.ph@gmail.com; 7 Department of Hepatobiliary Surgery, Vietnam-Germany Hospital, Hanoi 100000, Vietnam; trandinhthovd68@yahoo.com; 8 Department of Immunology and Allergy, National Otolaryngology Hospital, Hanoi 100000, Vietnam; vuminhthuc2010@yahoo.com.vn; 9 Biomedical Global Institute of Healthcare Research & Technology (BIGHEART), National University of Singapore, Singapore 117599, Singapore; ciezwm@nus.edu.sg; 10 Department of Psychological Medicine, Yong Loo Lin School of Medicine, National University of Singapore, Singapore 119228, Singapore; pcmrhcm@nus.edu.sg

**Keywords:** online food, food safety, Vietnam

## Abstract

This study aimed to examine: (1) how the Internet has changed consumers food-buying behavior and identify its associated factors; (2) consumers’ concern about food safety information of online food products. A cross-sectional study was performed from October to December 2015 in Hanoi—a Vietnamese epicenter of food service. One thousand seven hundred and thirty six (1736) customers were randomly chosen from food establishments of 176 communes. Data were collected through face-to-face interviews using structured questionnaires. The majority of participants reported using the Internet to search for food products (81.3%). The most crucial factors influencing food purchases through the Internet were convenience (69.1%) and price (59.3%). Only one-third of participants selected products based on accurate evidence about food safety certification or food origin. The majority of participants were concerned about the expiration date (51.6%), while brand (9.8%) and food licensing information (11.3%) were often neglected. People who were:(1) female, (2) highly influenced by online relationships, and (3) having difficulty in doing usual activities were more likely to look for online food products. These findings produce practical advice to consumers when purchasing their desired food products on the Internet, to online food retailers and to the Government of Vietnam to implement appropriate legislation regarding trading online food products.

## 1. Introduction

The recent development of the Internet has boosted the extension of online food services by enabling people to search, compare prices and conveniently access these services [1,2,3]. As of 2016, approximately 95% of the United States population searched for online food service information at least once [4], and while in 2015 more than one-third of Asia-Pacific participants, especially in developing countries, answered that they looked for and ordered products via the Internet [5]. However, such business raises food safety and hygiene considerations, particularly in low and middle—income countries where food systems are heterogeneous and fragmented, with a predominance of small informal food retailers [6]. Specifically, uncontrolled food processing, packaging and transferring among small online food retailers can elevate the risk of food contamination and contribute to food poisoning outbreaks due to the development of several pathogenic bacteria [7]. To strictly control food sold over the Internet, the Food Standards Agency in the United Kingdom has provided guidance which includes practical advice to customers, to businesses selling food online as well as to local authorities. In addition, in China, food safety practices through online food vendors came into the spotlight due to the report of foodborne illness incidents which stemmed from the microbiological risk of online food [8,9]. Therefore, the government of China has published the amendments to the Implementing Regulations of the Food Safety Law enforcing online food businesses to monitor food and carry out food examinations to ensure that the online food products are legally sourced, safely stored and transferred [10]. 

Vietnam is acknowledged as being among the countries with the highest growth rates of Internet penetration in Asia, leading to an undeniable development of the e-commerce market [11]. The number of Vietnamese customers purchasing products via the Internet or online social networking sites has been rising rapidly, and is projected to grow by 22 percent in 2017 and 13.2 percent by 2020 [12,13]. It has to be recognized that ordering food through social media platforms is a fast-growing and emerging trend in Vietnam, in which customers can buy handmade processed food provided by internet users who are processing, advertising and selling their products [14]. This upward trend has been fueled by the rapid expansion of Facebook and other social media users in Vietnam [15]. 

However, this kind of food trading also contains several drawbacks. The traditional face-to-face approach generates opportunities for consumers to subjectively discern the quality of the food products and the level of hygiene [16,17,18,19]. Meanwhile, the information of food products on the Internet is mostly self-advertised [20] which makes it difficult to verify the truth of the information about food traceability, food processing as well as identification of the trader’s permits [21,22,23]. In addition, the current food safety chain management still appear flawed [24], the Vietnam food administration are still lacking policies and e-commerce regulations in managing food services through online systems, particularly on new trendy trading methods such as social media [25]. Thus, it comes along with difficulty in guaranteeing food safety of online food purchase, which is less recognized in previous studies. 

Since regulations have not existed until recently, understanding consumer behaviors toward online food trading is essential to managing and recommending further actions. Restricting the dangers from food products purchased through an online trading platform is primarily relying on the cautiousness of online purchasing behaviors of customers. Existing literature indicated that one of the most requisite factors influencing food service selection was the reviews or suggestions of peers [26,27]. Nevertheless, the effects of online personal interaction on seeking food services via the Internet have not been investigated yet. This current study, therefore, aimed to determine the prevalence of people who would take advantages of the Internet to purchase food products and identify its associated factors. Our study also provided more insightful understanding regarding how consumers are concerned about food safety information of online food products.

## 2. Materials and Methods 

### 2.1. Study Setting, Sample Size, and Sampling Method

A cross-sectional study was performed from October to December 2015 in Hanoi—a Vietnamese epicenter of food service. In this study, we randomly selected 176 communes from 29/30 districts in Hanoi. In each commune, we randomly selected food facilities based on the list of food facilities which are businesses registered with local governmental agencies. Participants were randomly chosen from each identified facility. The purpose of the study was clearly explained to all participants and participant information sheets were provided. The participants were required to meet the following inclusion criteria: (1) being 15 years old or above; (2) agreeing to participate in the study and (3) having the capacity to answer questions. A total of 1736 participants agreed to enroll in this study and the response rate was 99.2%. To determine the reliability as well as the acceptability of questionnaire, a pilot survey was conducted among 50 customers of various genders and ages. As a result minor changes were made to the official questionnaire. 

### 2.2. Measures and Instruments

Data were collected through face-to-face interviews using structured questionnaires. Interviewers were students who enrolled in the Master of Public Health Program at the Hanoi Medical University. A total of 92 students were divided into nine groups and each group was under supervision of a professor. The interviews were conducted inside the food facilities and it took 20 min to finish the interview. The variables of interest are listed below. 

#### 2.2.1. Socioeconomic Characteristics

Socioeconomic data including age, gender, education attainment, marital status, employment, and monthly income were collected. 

#### 2.2.2. Online Shopping Behavior of Customers

In this study, the definition of utilizing online food services was when consumers use online technology to find and purchase their desired food products. Participants were asked about their experiences purchasing food products that they searched on the Internet and the main type of online platform used to search for food products. In terms of food safety and hygiene information, participants were asked about the most important factor influencing customer behaviors in purchasing online food products, the perceived level of trust in food hygiene information and the information on the label of ready-to-eat food which was of concern.

#### 2.2.3. Influences of the Online Interpersonal

The study also identified factors associated with using the Internet in seeking food services. Respondents were asked about online interpersonal influences using a 5-point Likert scale (For example, the measure included the following questions with five response options: Always/High influence; Usually/Moderate influence; Sometimes/Some influence; Rarely/Little influence Never/No influence). The used questions are showed below:

1. How much do the images and information about behavior and lifestyle shared by your friends on the Internet influence your own behavior and lifestyle?
2. How often do you visit spots that were liked, recommended and posted by your friends on the Internet?
3. How often do you participate in activities that were liked, recommended and posted by your friends on the Internet?

#### 2.2.4. Other Characteristics

In this study, health problems was examined by using EuroQol—dimensions—five levels (EQ-5D-5L). The EQ-5D-5L included five dimensions: Pain/Discomfort, Anxiety/Depression Mobility, Self-care and Usual Activities with five levels of response: no problems, slight problems, moderate problems, severe problems, and extreme problems. Body-mass index was calculated based on height (cm) and weight (kg) of participants. We use the modified BMI categorization for Asian population which recommended by World Health Organization (WHO) with the cut-off point: underweight (<18.5 kg/m^2^), normal weight (18.5–22.9 kg/m^2^), overweight (23–24.9 kg/m^2^), obesity (≥25 kg/m^2^) [28].

### 2.3. Statistical Analysis

STATA software version 12.0 (StataCorp. LP, College Station, TX, USA) was employed to analyze the data. Independent *T*-test and X^2^ tests were used to examine the differences in characteristics between people using and not using the Internet to seek food products. Multivariate binominal logistic regression was performed to identify the factors associated with food product selections via the Internet. Forward stepwise selection strategy was utilized to remove non-significant factors, with the *p*-value of log likelihood ratio test set as less than 0.2 and this was the threshold to include a variable. The statistical significance was determined with a *p*-value < 0.05.

### 2.4. Ethics Approval 

The study was approved by the Institutional and Review Board (IRB) of Hanoi Branch of Food Safety at The Hanoi Health Department (code: 06/CCATVSTPHN), based on research ethics regulations of the Ministry of Health. Written informed consent was obtained from all participants after detailed explanation. Participants were allowed to withdraw from the interview at any time and it would not affect their usage of food services. All subjects’ information was kept confidential and anonymous data were only used for analysis. 

## 3. Results

The demographic and socioeconomic characteristics of participants and their behavior in using the Internet for seeking food services is given in Table 1. A total of 1298 participants used the Internet, 61.1% were female and the number of females seeking online food services was significantly higher than number of male that did (84.9% and 75.6% respectively). Most of the respondents had high school education and above. In all, 35.9% were office workers and 21.3% were students. The total average monthly income was 5.4 million VND (SD ± 5.6).

Attitudes and behaviors of consumers towards online food information are illustrated in Table 2. Out of 1298 participants using the Internet, the majority of participants (80%) sought food products via the Internet, social media was the most popular online platform (59.8%). 

The factors that majorly influenced consumer behavior in purchasing online food products was convenience (69.1%), followed by price (59.3%). Approximately 46% of respondents selected food products based on their self-assessment in food hygiene, and the percentage of consumers who referred to food hygiene and safety certification was the smallest, accounted for only 30.4%. Nevertheless, only one-third of participants believed in the food safety information provided online (37.7%). The ingredients were the most important requisite criteria in ordering ready-to-eat food (52.2%). Regarding food labels, participants were most concerned about expiration date and brand (51% and 22.2%). Only approximately 10% of participants were interested in production facilities’ name as well as food license information.

Table 3 compares how common using the Internet to seek food products was according to multiple forms of interpersonal influences on behaviors, lifestyles and social activities. People who were highly influenced in behaviors and lifestyle by online relationships were more likely to purchase food products through the Internet (97%, *p* < 0.01). Furthermore, seeking online food products was more common among customers who always visited entertainment establishments (94.8%, *p* < 0.01) and always engaged in activities (97.1%, *p* < 0.01) recommended by their friends on the Internet.

Table 4 compares the occurrence of health problems and BMI index between participants purchasing and not purchasing food products via the Internet. Respondents who suffered from pain/discomfort (*p* < 0.01), anxiety/depression (*p* < 0.01), had difficulty in mobility (*p* = 0.03) and problems with performing daily routines (*p* < 0.01) were more likely to use the Internet to search for food products, compared to counterparts. 

The factors related to using the Internet in seeking food services examined by the reduced multivariate logistic regression are shown in Table 5. 

People who were female (OR = 1.87, 95% CI = 1.36–2.58) and who lived with spouse or partner (OR = 1.42, 95% CI = 1.02–1.97) were more likely to search for online food products. Using the Internet purposely to search for food services was also positively associated with a self-perceived high level of influence on behaviors and lifestyle from online interpersonal relationships (OR = 5.5, 95% CI = 1.91–15.81), high frequency of visiting places (OR = 1.75, 95% CI = 1.24–2.48) and engaging in activities which were recommended by friends on the Internet (OR = 5.86, 95% CI = 2.02–16.96). In addition, a higher proportion of seeking food products via the Internet was associated with people undergoing pain or discomfort (OR = 2.57, 95% CI = 1.06–6.24) and having difficulty in doing usual activities (OR = 2.29, 95% CI = 1.41–3.7). 

## 4. Discussion

To our knowledge, this study is one of the first of its type in Vietnam assessing food information and consumers’ behaviors towards food product selections purchased via the Internet. The majority of participants reported a positive attitude towards using the Internet in seeking food services. However, a low proportion of customers was chiefly concerned about the food safety of food products which they searched on the Internet. Only one-third of participants trusted the food safety and hygiene of food products provided online. In addition, people who were female, highly influenced by their online peers, suffered from pain or discomfort, and having difficulty in performing usual activities were significantly associated with Internet usage to seek food services. 

In this study, in using the Internet group, we found that approximately 80% of consumers accessed food products via the Internet, which is consistent with previous studies [4,29]. With the fast-paced environment today, the Internet has played an integral part in seeking various types of service information [30] and helped users to reduce cost and time to acquire information [2,11]. In addition, our findings also suggested that online social networks have become one of the most common sources to seek out food products. This is in line with previous studies, which reported that food businesses on social network sites are being accessed by a large number of food consumers [31,32]. This phenomenon may be due to the exponential growth in the number of social media users [33] and how social media assists individuals in expanding their communication network [34]. 

In our study, the number of consumers who tended to select food products based on the price and convenience was the largest, while those based on certified food hygiene and safety certification was the smallest. This is an expected finding because competitive price and convenience are the two main concerns in Vietnamese online shopping culture [35]. In context of Vietnam, consumers suppose that food hygiene is a requisite factor which they use to restrict the number of services to look at, while price and convenience are two factors among the most decisive factors which influence behavior of choosing food products on the Internet [36,37]. A study by Nguyen also reveals that a high percentage of customers used calories and nutrition labeling for ordering food [38]. In addition, the percentage of consumers trusting in food hygiene information of online food products was 37%, lower than a study conducted among Australian consumers [39]. The literature suggested that the number of people who distrusted online food information had increased because they might encounter potentially inaccurate and misleading information [20,40]. These concerns from consumers are valid due to the fact that distributors can easily advertise their food items [41], and most of these websites do not undergo the traditional mechanism of accuracy verification [20,42]. For traditional food establishments, consumers can subjectively assess food safety through food processing and aesthetic value. By contrast, for online food services, clients can only decide based on advertising footage as well as reviews from other customers, which is hard to verify because of its virtual nature. 

In terms of ready-to-eat food, the participants were primarily concerned about expiration date on the food label, but the information about facilities or food license were often neglected which may raise a question regarding unhygienic conditions or microbiological quality [43]. Moreover, consumers often relied on their own judgment of food hygiene rather than accurate evidence of food safety certification or food origin. Their misjudgment could be due to the lack of food safety information on labels, especially on online products [44,45]. We hypothesize that consumers can only rely on self-advertising information, subjective assessment or, more evidently, expiration date to confirm the food safety conditions of these products [46]. Hence, consumers are misled by advertising information on the Internet, which cannot be verified by relevant authorities [47], raising the concern of food safety and foodborne diseases. 

Our current study also suggested that seeking online food products among participants was greatly influenced by online interactions, especially by peers. These are expected findings which can be explained by high interact-ability that has been brought by the Internet. When peers share, post illustrating pictures or reviews about food products on social media, those who are more easily influenced by others would trust their friends’ selection and experiences [31,48]. It would be beneficial in case the source of data is credible and verified by relevant authorities [49]. On the other hand, it would be unprofitable if the opinions from peers are unjustified and biased [50]. As a result, this kind of interaction is both impulsive and a barrier to ensuring food safety. Therefore, verifying online food product information by credible sources is imperative in order to ensure food safety. 

Moreover, in this study, women were more likely to purchase food products through the Internet and this result can be explain by differences in gender roles in Vietnamese families. Women have to bear most of responsibilities regarding household chores, especially preparing meals and food shopping [51,52,53]. Therefore, enormous numbers of women are approaching to the Internet to seek food products, which help them to achieve time efficiency [54]. A higher likelihood of using the Internet to purchase food products can be seen among people suffering from pain or discomfort and having problems in performing daily activities. Given the fact that they are limited to involve in instrumental activities of daily living such as doing housework, grocery shopping or preparing meals, they do not have abilities to live independently and have to depend on others for help [55]. Thus, they use the Internet as a useful tool that strongly supports them in fulfilling their tasks, for instance seeking food service information, ordering food products [56]. 

### 4.1. Implications of the Study

Our study highlights the necessity for consumers to carefully discern online food product information instead of mainly relying on subjective assessment. It is critical for consumers to consider the origin of food, food processing, and appropriate certification granted by relevant authorities prior to purchasing online food products. Besides, in order to increase consumer’s trust, food retailers should also proactively provide accurate and sufficient information about their food products online. To help customers to gain precise information about online food products, the Government should require the online food retailer to register their business and provide information related to food safety. All the information will be entered into a database system which should be regulated by the Government. This system already proved to be a successful strategy in China [10]. Consumers can refer to this system to select online food services and report violations if they have complaints about food safety standards.

Our findings also indicate that strategies should focus on the online community due to the high interaction between online customers. The enforcement of potential interventions through online campaigns is necessary to raise awareness about food hygiene and safety. Our study suggests that interventions should target on women who are primarily responsible for planning family meals as well as those who have difficulty in performing daily activities, in order to provide proper safety information of online food services. 

### 4.2. Strengths and Limitations

The strength of this study was the large sample size across several levels of food services in 29 rural and urban districts in Hanoi–a center of diverse food services in Vietnam. Nonetheless, several limitations need to be considered. First, the data were based on self-reports, which may lead to recall bias. Second, the causal relations between routine Internet usage and level of online interpersonal interactions influence could not be investigated due to the nature of cross-sectional study. We cannot conduct more appropriate research due to limited funding. In addition, there is very little literature related to this topic. Thus, our findings could be used as foundation for future studies. Third, we only recruited participants in Hanoi; hence, the sample were not representative of general population and limiting the generalizability of study. Finally, this study did not include other variables such as history of food poisoning after using online food services and how consumers verify the accuracy of food services information. Future studies should examine association between utilization of food services online and foodborne illnesses.

## 5. Conclusions

In conclusion, our study emphasized that using the Internet in seeking food service information was a common practice among people living in Hanoi, Vietnam and online interpersonal influences took a fundamental part. A high percentage of consumers were unconcerned about accurate evidence regarding food safety in selecting food products on the Internet. The conclusion of our findings produces practical pieces of advice to consumers buying online food, to food retailers selling food over the Internet and to the Government of Vietnam to implement appropriate legislation regarding online food product information.

## Figures and Tables

**Table 1:** Socioeconomic characteristics of participants seeking food products via the Internet in Hanoi in 2015.

Characteristics	Using the Internet in Seeking Food Products		Not Using the Internet in Seeking Food Products		Total		*p*-Value
	*n*	%	*n*	%	*n*	%	
**Gender**							
Male	380	75.6	123	24.4	503	38.9	<0.01 *
Female	672	84.9	120	15.1	792	61.1	
**Marital status**							
Single	412	80.3	101	19.7	513	39.6	0.66 *
Live with spouse/partner	628	82	138	18	766	59.1	
Divorced/widow	13	76.5	4	23.5	17	1.3	
**Education**							
Under High school	75	78.1	21	21.9	96	7.4	0.70 *
High School	286	82	63	18	349	27.2	
College, University	680	81.1	159	18.9	839	65.4	
**Employment**							
Worker	140	76.5	43	23.5	183	14.1	0.10 *
Office workers	388	83.3	78	16.7	466	35.9	
Students	225	81.2	52	18.8	277	21.3	
Housewife	114	86.4	18	13.6	132	10.2	
Other jobs	187	78.2	52	21.8	239	18.5	
**Age group**							
15–24	304	82.2	66	17.8	370	28.9	0.81 *
25–34	369	80	92	20	461	36	
35–44	196	81.3	45	18.7	241	18.8	
45–54	104	83.2	21	16.8	125	9.7	
55–64	57	82.6	12	17.4	69	5.4	
65+	9	69.2	4	30.8	13	1.2	
	**Mean**	**SD**	**Mean**	**SD**	**Mean**	**SD**	
**Average monthly income (million VND)**	5.3	5.1	5.7	7.6	5.4	5.6	0.9 **

Number of total participants, *n* = 1298. Statistical significance level *p* < 0.05 by * Chi-square test, ** Mann Whitney test.

**Table 2:** Attitudes and behaviors towards food information provided online among participants in Hanoi (Vietnam) in 2015.

Attitude and Behavioral Attribute	*n*	%
**Using the Internet (*n* = 1736)**	1298	74.8
**Using the Internet in seeking food products (*n* = 1298)**	1055	81.3
**Preferred online platform in seeking food products (*n* = 1055)**		
Website	298	34.9
Social media	511	59.8
Others	45	5.3
**Factors mostly influencing customer behaviors in purchasing online food products (*n* = 1055)**		
Convenience	714	69.1
Price	622	59.3
Hygiene by self-assessment	465	46.0
Reference by friends	379	36.5
Certified—food hygiene facilities and food source	319	30.4
**Level of trustworthiness regarding hygiene information of online food products (*n* = 1055)**		
Unbelievable	49	4.9
Neutral	574	57.4
Believable	377	37.7
**The most important criteria to order online ready-to-eat food (*n* = 1055)**		
Products’ name	451	45.4
Ingredients	525	52.2
Advertisement	212	21.1
Price	442	42.5
**The most concerned information on the label of ready-to-eat food (*n* = 1055)**		
Products’ name	229	22.2
Production facilities’ name	95	9.2
Business license	120	11.0
Date of manufacture	59	5.7
Expiry date	526	51.0

**Table 3:** Association between online interpersonal influences and seeking food products on the Internet in Hanoi (Vietnam) in 2015.

Influencing Attribute	Using the Internet in Seeking Food Products		Not using the Internet in Seeking Food Products		Total		*p*-Value *
	*n*	%	*n*	%	*n*	%	
**Self-perception of the influences of online relationships on behaviors and lifestyles**							
Little or no influence	601	76.4	186	23.6	787	62.7	<0.01 *
Some influence	288	86.5	45	13.5	333	26.5	
High influence	131	97.0	4	3.0	135	10.8	
**Visiting entertainment establishments recommended by online friends**							
Rare or never	418	74.6	142	25.4	560	45.6	<0.01 *
Frequently	460	86.1	74	13.9	534	43.5	
Always	127	94.8	7	5.2	134	10.9	
**Engage in activities recommended by online friends**							
Rare or never	466	76.9	140	23.1	606	49.0	<0.01 *
Frequently	421	85.0	74	15.0	495	40.0	
Always	132	97.1	4.0	2.9	136	11.0	

Number of total participants, *n* = 1298. Statistical significance level *p* < 0.05 by * Chi–square test.

**Table 4:** Differences in health problems and BMI index between participants using and not using the Internet to seek food products.

Characteristics	Using the Internet in Seeking Food Products		Not using the Internet in Seeking Food Products		Total		*p*-Value *
	*n*	%	*n*	%	*n*	%	
**BMI**							
Underweight	36	80.0	9	20	45	3.6	0.97
Normal weight	785	80.9	185	19.1	970	78.5	
Overweight	171	79.5	44	20.5	215	17.4	
Obesity	4	80.0	1	20	5	0.5	
**Pain/discomfort**							
No problem	920	79.9	232	20.1	1152	89.4	<0.01 *
Having problems	127	92.7	10	7.3	137	10.6	
**Anxiety/Depression**							
No problem	849	79.3	222	20.7	1071	84.8	<0.01 *
Having problems	173	90.1	19	9.9	192	15.2	
**Difficulty in mobility**							
No problem	954	80.2	235	19.8	1189	94.7	0.03 *
Having problems	60	90.9	6	9.1	66	5.3	
**Difficulty in self-care**							
No problem	1010	81.7	226	18.3	1236	97.7	0.89 *
Having problems	24	82.8	5	17.2	29	2.3	
**Difficulty in doing usual activities**							
No problem	740	79.2	194	20.8	934	74.7	<0.01 *
Having problems	277	87.7	39	12.3	316	25.3	

Number of total participants, *n* = 1298, Statistical significance level *p* < 0.05 by * Chi–square test.

**Table 5:** Factors associated with consumer’s preferences regarding purchasing online food products in Hanoi (Vietnam) in 2015.

Characteristics	Using the Internet in Seeking Food Products	
	95%CI	OR
**Gender**		
Male	Ref	-
Female	1.87 ***	1.36; 2.58
**Marital status**		
Single	Ref	-
Live with spouse/partner	1.42 **	1.02; 1.97
**Education**		
Under High school	Ref	-
High School	1.29	0.90; 1.86
**Self-perception of the influences of online relationships on behaviors and lifestyles**		
Little or no influence	Ref	-
Some influence	1.48 *	0.98; 2.24
High influence	5.50 ***	1.91; 15.81
**Visiting entertainment establishments recommended by online friends**		
Rare or never	Ref	-
Frequently	1.75 ***	1.24; 2.48
**Engage in activities recommended by online friends**		
Rare or never	Ref	-
Always	5.86 ***	2.02; 16.96
**Pain/discomfort**		
No problem	Ref	-
Having problems	2.57 **	1.06; 6.24
**Difficulty in mobility**		
No problem	Ref	-
Having problems	3.27	0.74; 14.34
**Difficulty in self-care**		
No problem	Ref	-
Having problems	0.08 ***	0.02; 0.43
**Difficulty in doing usual activities**		
No problem	Ref	-
Having problems	2.29 ***	1.41; 3.70
**Constant**	1.17	0.83; 1.65

* Indicate significance level *** *p* < 0.01, ** *p* < 0.05, * *p* < 0.1 by multivariate binominal logistic regression. OR: Odds Ratio; 95% CI: 95% Confidence Interval. Ref: reference group.

## Data Sharing Statement

The data that support the findings of this study are available from Institute for Preventive Medicine and Public Health, Hanoi Medical University but restrictions apply to the availability of these data, which were used under license for the current study, and so are not publicly available. Data are however available from the authors upon reasonable request and with permission of from Institute for Preventive Medicine and Public Health, Hanoi Medical University.
